# Genomic Variability and Post-translational Protein Processing Enhance the Immune Evasion of *Mycoplasma hyopneumoniae* and Its Interaction With the Porcine Immune System

**DOI:** 10.3389/fimmu.2020.510943

**Published:** 2020-10-07

**Authors:** Gaojian Li, Enoch Obeng, Jinqi Shu, Jianhong Shu, Jian Chen, Yuehong Wu, Yulong He

**Affiliations:** ^1^Department of Biopharmacy, College of Life Sciences and Medicine, Zhejiang Sci-Tech University, Hangzhou, China; ^2^Zhejiang Hom-Sun Biosciences Co., Ltd., Shaoxing, China

**Keywords:** *Mycoplasma hyopneumoniae*, genomic variability, immune evasion, immune interaction, post-translational protein processing

## Abstract

*Mycoplasma hyopneumoniae* (*M. hyopneumoniae*, Mhp) is a geographically widespread and economically devastating pathogen that colonizes ciliated epithelium; the infection of Mhp can damnify the mucociliary functions as well as leading to *Mycoplasma* pneumonia of swine (MPS). MPS is a chronic respiratory infectious disease with high infectivity, and the mortality can be increased by secondary infections as the host immunity gets down-regulated during Mhp infection. The host immune responses are regarded as the main driving force for the disease development, while MPS is prone to attack repeatedly in farms even with vaccination or other treatments. As one of the smallest microorganisms with limited genome scale and metabolic pathways, Mhp can use several mechanisms to achieve immune evasion effect and derive enough nutrients from its host, indicating that there is a strong interaction between Mhp and porcine organism. In this review, we summarized the immune evasion mechanisms from genomic variability and post-translational protein processing. Besides, Mhp can induce the immune cells apoptosis by reactive oxygen species production, excessive nitric oxide (NO) release and caspase activation, and stimulate the release of cytokines to regulate inflammation. This article seeks to provide some new points to reveal the complicated interaction between the pathogen and host immune system with Mhp as a typical example, further providing some new strategies for the vaccine development against Mhp infection.

## Introduction

*Mycoplasma hyopneumoniae* (*M. hyopneumoniae*, Mhp) is one of the primary pathogens of *Mycoplasma* pneumonia of swine (MPS), also known as enzootic pneumonia (EP) (1). This chronic respiratory pathogen mainly leads to decreased daily gain weight and serious clinical symptoms in growing and finishing pigs. MPS is reported worldwide and causes significant economic losses, mainly from the treatment, immunization, control/eradication and increased mortality caused by secondary infections (viruses, bacteria or parasites) (2). The *in vitro* culture of Mhp is strenuous because of its fastidious requirement for culture medium (3, 4), which limits the large-scale preparation and industrial production of bacteria-based vaccines. At present, commercial genetically engineered vaccines and other types of novel vaccines are still under development; vaccination (inactivated/attenuated vaccines) is the main method to control MPS, but it provides partial protection, and it cannot eliminate Mhp from farms or bodies completely (5).

Mhp is one of the smallest self-replicating microorganisms with less cell walls and reduced genome scale; these characteristics make Mhp rely on its surroundings (*in vivo*/*in vitro*) to acquire adequate nutrients like nucleic acid, lipid, and amino acid for its proliferation (6). Also, the limited genome size and protein expression scale allow Mhp to complicate its proteomics by Post-translational protein processing (6). Mhp infection is widespread in farms and also difficult to eliminate, which indicates a certain degree of immune evasion. However, a clear picture of the immune evasion mechanisms of Mhp and its interaction with host immune system is still missing.

In this review, we summarized the mechanisms of Mhp immune evasion from genomic variability and Post-translational protein processing. In addition, Mhp can induce the apoptosis of immune cells by reactive oxygen species (ROS) production, excessive nitric oxide (NO) release and caspase activation, and stimulate the release of cytokines to regulate inflammation. This article aims to use Mhp as an example to outline the comprehensive interaction between pathogen and the host immune system, and new strategies for the next-generation of efficient vaccine development for the control of MPS are also proposed.

## Genomic Variability and Post-translational Protein Processing Enhance the Immune Evasion Capacity of Mhp

### Genome Variability Diversifies the Protein Expression Profile and Cell Surface Antigens Appearance

**Figure 1:**
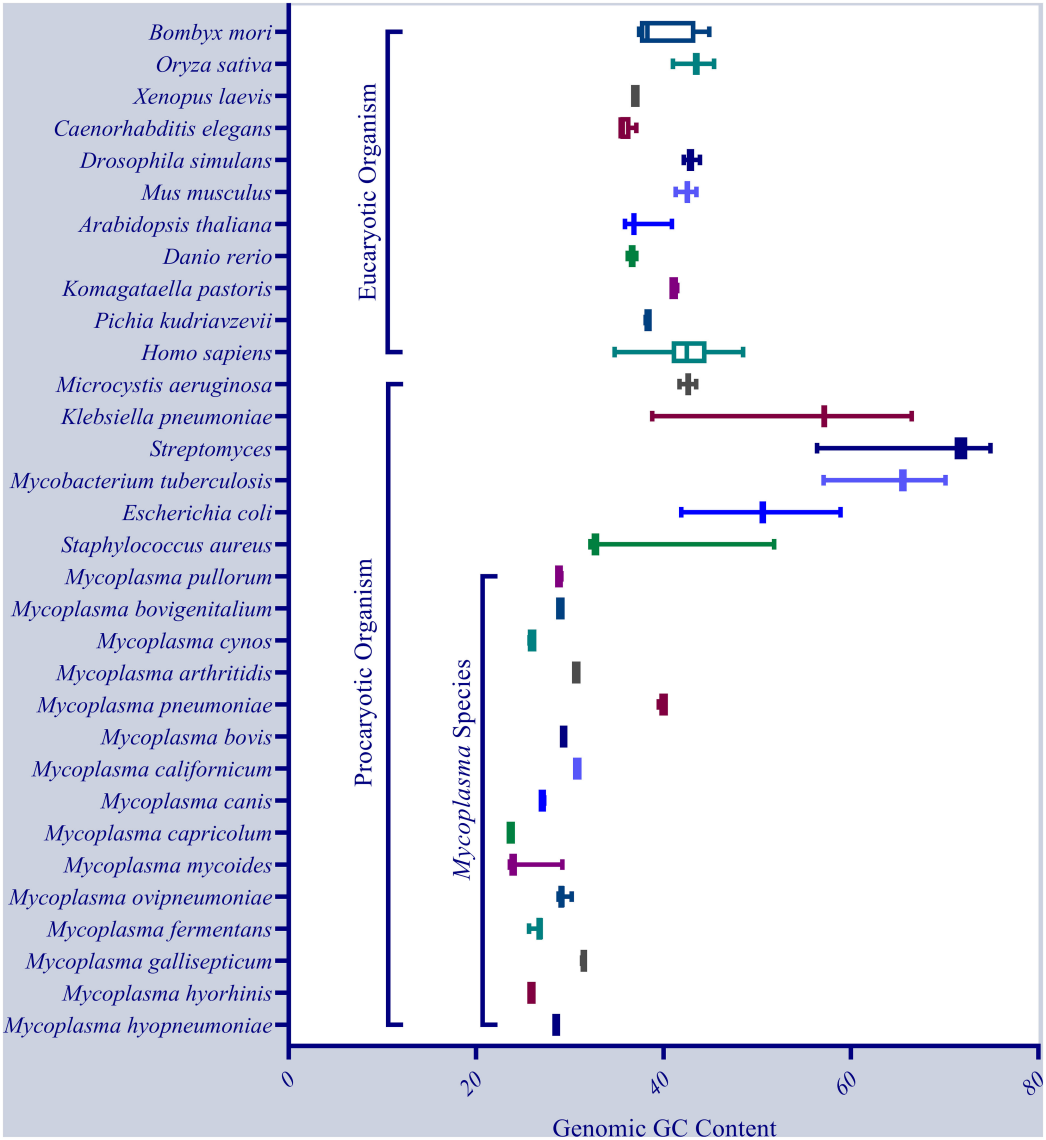
The comparison of genomic GC content between *Mycoplasma hyopneumoniae* and other species, indicating a low GC content in almost all *Mycoplasma* species except *Mycoplasma pneumoniae*.

The genomes of 12 Mhp strains (J, 7448, 232, 168, 168-L, 7422, KM014, TB1, 11, NCTC10127, ES-2, and F7.2C) were sequenced and uploaded into the database of the National Center for Biotechnology Information (NCBI). In general, the average genomic size of Mhp is 0.9199 Mb, encoding 744 genes and 645 proteins, the mean GC content is 28.54%, which is a significantly low value compared with other species. Figure 1 shows the genomic GC content of Mhp and other species; Supplementary Table 1 provides the detailed genomic GC content information of these species. Studies had reported that the GC-rich regions had more conservative mutation rate compared with the GC-poor regions (7). On the other hand, GC content is an important parameter for genome organization and gene expression (8, 9), the low GC content gives Mhp complex transcriptional organization, unique intrinsic terminator stem-loop formation and individual ribonuclease P (RNase P) structure (10, 11). Earlier studies demonstrated that *Mollicutes* genomes had increased recombination and mutation rate as the environment changed (12), and the high genomic diversity between different Mhp strains was reported in follow-up experiments (13).

Genomic diversity can be associated with discrepant pathogenicity of pathogens (14). A comprehensive genomic analysis was performed to evaluate the genomic variability between pathogenic strain Mhp 168 and its high-passaged attenuated strain Mhp 168-L. Some mutative coding sequences were found in Mhp 168-L including adhesins (P97, P102, P146, P159, P216, and LppT), cell envelope protein (P95), cell surface antigen (P36), secretory proteins, and chaperone protein (DnaK), which could be associated with their different pathogenicity (15). Results from other experimental methods also supported the genomic variability between Mhp strains, including multiple locus variable number tandem repeat analysis (MLVA) (13, 16–18), random amplified polymorphic DNA (RAPD) analysis, pulsed-field gel electrophoresis and nested polymerase chain reaction (nPCR) (19–22). This review (23) provides detailed information about the Mhp genomic classification and variability.

In brief, genomic variability of Mhp has been identified, and the effects for controlling MPS are summarized as follows. (1) Gene mutations in immunogens like P97 and P146 (both of P97 and P146 are important adhesins and commonly used as immunogens in genetically engineered vaccines) have a negative impact for the vaccine development (24, 25). (2) Antibiotic efficacy decreased with the mutations happened in topoisomerase enzymes (26–29) and 23S rRNA (30, 31). (3) Genomic variability increases the porcine susceptibility when infected by different Mhp strains.

### Complex Post-translational Protein Processing Contributes to the Immune Evasion

It is generally accepted that bacteria can use protein hydrolysis processing to accommodate the changing environment and manipulate host immune responses (32). Mhp cell surface proteins can be processed to play roles in cell growth, biofilm formation, peptide secretion and pathogenesis (33, 34). For bacteria with reduced genomes, proteolytic regulation allows them to produce novel proteoforms and digest host cell proteins for their synthesis (35). It is reported that the lipid-associated membrane proteins (LAMPs) from Mhp are necessary for cell adhesion, infection, disease development, and immunosuppressive effect (36). For example, a multifunctional adhesin mhp390 can induce apoptosis in different immune cells and exacerbate inflammatory responses (37). Besides, two surface-exposed Mhp proteases putative Xaa-Pro aminopeptidase (MHJ_0659; PepP) and oligoendopeptidase F (MHJ_0522; PepF) share substrates and participate in the cleavage of many biologically active proteins (bradykinin, substance P, neurokinin A, and neuropeptide Y) for the regulation of lung homeostasis (38).

The Post-translational protein processing events in Mhp proteomics can expand protein functions and expose more epitopes (39), which may increase the host immune burden. Protein cleavage events were reported in adhesins (25, 33, 34, 40–46), lipoproteins (6) as well as the surface moonlighting proteins (6, 39, 47, 48) (Table 1). Besides, plasminogen is an available host extracellular component in the respiratory tract, which can repair damaged tissues and regulate inflammatory responses (50, 51). Studies had found that during the experimental Mhp infection, plasmin activity was enhanced as a part of the host immune responses, and vaccination could weaken this effect (52). In this process, plasminogen can be activated by P97/P102 adhesion family proteins (53), P116 (44), glutamyl aminopeptidase (GAP; MHJ_0125) (47), leucine aminopeptidase (LAP; MHJ_0461) (54), and fructose-1, 6-bisphosphate aldolase (FBA) (55). The plasminogen activation can improve the Mhp adhesion efficiency and stimulate cytokines release like tumor necrosis factor α (TNF-α), interleukin (IL)-1β, and IL-6 (52), these may be secreted by endothelial cells, monocytes, macrophages, and dendritic cells (DCs) (56).

**Table 1:** Protein processing events reported in Mhp proteins.

**Protein**	**Gene**	**Cleavage site**	**Products**	**Function**	**Reference**
P146	MHJ_0663 (Q4A925)	^350^KTY↓AE^355^ ^672^ATEF↓QQ^677^	P50_P146_, P40_P146_, P85_P146_	Adhesin, bind epithelial cilia and biotinylated heparin	(25)
Lipoprotein P65	MHJ_0656 (Q4A932)	^4^TTE↓NWL^109^ ^165^LTM↓SVG^170^ ^360^TNF↓DDF^365^ ^501^VAF↓FAE^506^	Recombinant protein fragments (37.9 kDa, 29.9 kDa, 54.2 kDa, 52.3 kDa, 30.4 kDa, 16.3 kDa)	Lipolytic enzyme	(6)
Membrane protein P159	MHJ_0494 (Q4A9J1)	^233^F↓Q^234^ ^981^F↓Q^982^	P27, P110, P52	Cilium adhesin	(40)
Membrane protein P216	MHJ_0493 (Q4A9J2)	^1072^TNF↓QE^1076^	P120, P85	Cilium adhesin	(41, 42)
Mhp384	MHJ_0368 (Q4A9W5)	^527^ILF↓NEE^532^	P60_384_, P50_384_	Cilia and heparin adhesin	(43)
Mhp385	MHJ_0369 (Q4A9W4)	-^761^LNV↓AVS^766^	P115_384_, P88_384_, P27_384_	Cilia and heparin adhesin	(43)
P116	*mhp108*	^295^K↓W^296^, ^541^A↓I^542^ ^699^L↓J^700^	Recombinant protein fragments F1 (33.5 kDa), F2 (32.5 kDa), F3 (21.8 kDa), F4 (39.6 kDa)	Porcine fibronectin, plasminogen, and respiratory cilia adhesin	(44)
Mhp107	*mhp107*	^345^T↓E^346^, ^681^Q↓G^682^	Recombinant protein fragments F1 (42.7 kDa), F2 (42.1 kDa), F3 (44.8 kDa)	Porcine heparin, fibronectin, and plasminogen adhesin	(49)
Protein containing the P97 domain	MHJ_0194	^−^	Recombinant protein fragments (22, 28, 66, 94 kDa)	Cilium adhesin	(33)

MIB–MIP is a mycoplasmal system that captures and cleaves immunoglobulin (Ig) G, which is mediated by *Mycoplasma* immunoglobulin binding protein (MIB) and *Mycoplasma* immunoglobulin protease (MIP), respectively. The MIB-MIP system had been reported in *Mycoplasma mycoides* subspecies *capri*, and the multiple copies of MIB-MIP genes were also found in Mhp genome (57), while the role of the MIB-MIP system in the interaction between Mhp and host immune system still needs further studies.

In short, as a genome-reduced pathogen, Post-translational protein processing and genomic variability complicate the Mhp cell surface antigen appearance and expand protein functions, which may aggravate disease development and regulate lung homeostasis to promote the proliferation of Mhp. For the immune system, abundant exposed epitopes can affect the Pathogen-Associated Molecular Patterns (PAMPs) to reduce immune efficiency, and more functional virulence factors can contribute to the negative regulation of the immune system.

## The Interaction Between *Mycoplasma hyopneumoniae* and Host Immune System

**Figure 2:**
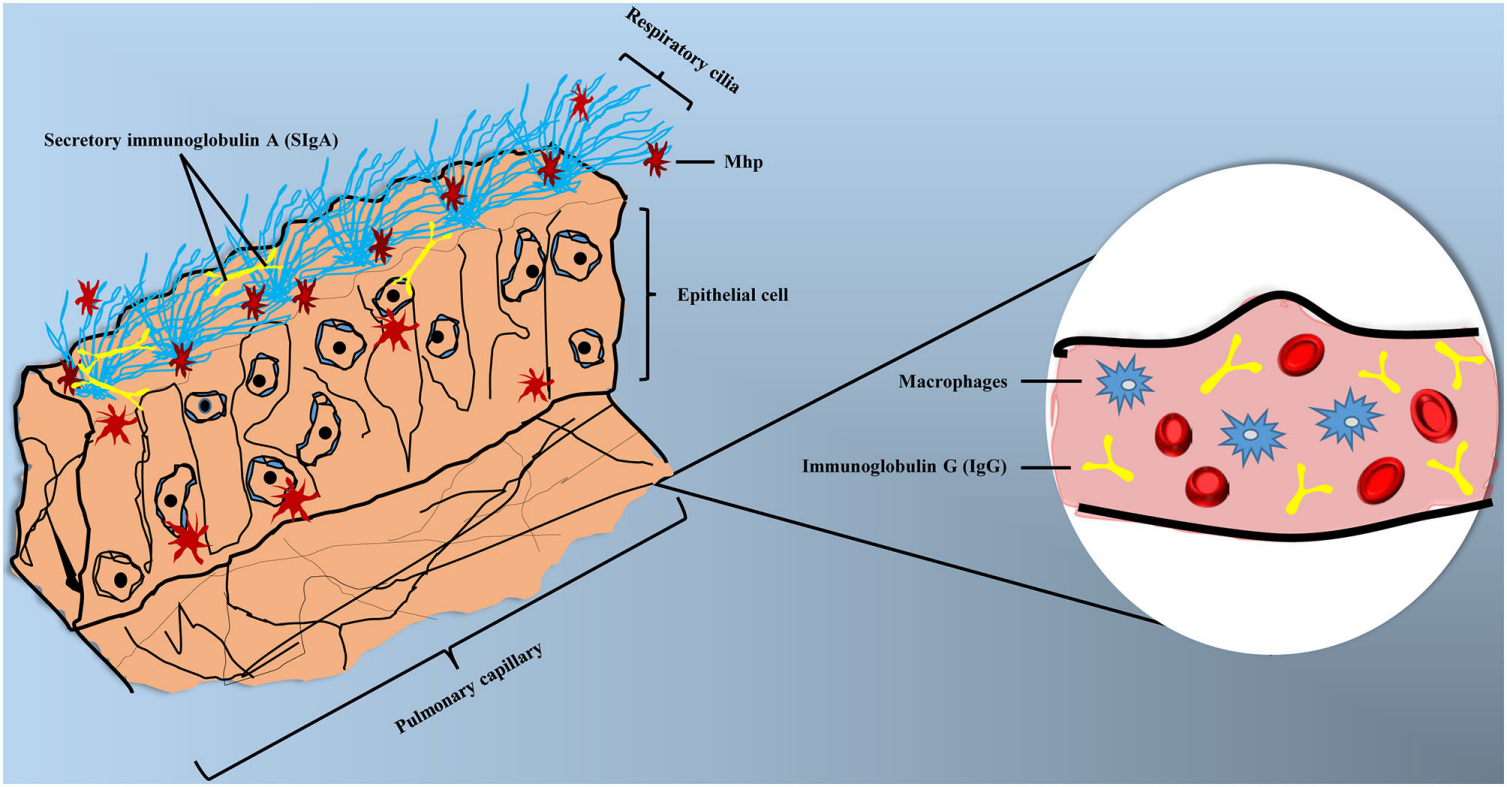
Partial pattern diagram of Mhp infection. Mhp infection destroys the cilium structures, leading to the apoptosis of epithelial cells, as well as increasing the secondary infection risk. Mhp can survive inside the epithelia cells and move to other tissues with disease development. The production of secretory IgA (SIgA) and IgG and phagocytic action of macrophage are involved in the immune responses.

Different immune cells can form a complex and sophisticated network to recognize pathogens and provide direct defense against their invasion. In the respiratory tract, the mucosal immune system is constructed by epithelial barriers, mucosal dendritic cells, innate lymphoid cells, natural killer cells, natural killer T cells, mast cells, and eosinophils (58). Mhp can invade organisms by endotracheal, intranasal or aerosol, while the endotracheal is the most effective method to induce MPS experimentally (59). Thereafter, Mhp can adhere to the cilium surface and spread through the respiratory tract, destroying cilium structures and causing apoptosis to epithelial cells (60). Clinical symptoms of MPS can be summarized as dry coughing, dyspnea, inflammation response, and immunosuppression (61, 62), the accumulation of mononuclear leukocytes in bronchioles and perivascular was also reported (63). Partial pattern diagram of Mhp infection is shown in Figure 2, Mhp infection destroys cilium structures and induces the apoptosis of epithelial cells, which may increase the risk of secondary infections. Mhp can survive inside the epithelia cells and move to other tissues with disease development. The production of secretory IgA (SIgA), IgG, and phagocytic action of macrophage are involved in the immune responses.

### Pulmonary Macrophage Cells (PAMs) Responses During Mhp Infection

#### PAMs Can Recognize Mhp and Release Cytokines

Studies had reported that PAMs could recognize and provide immune defense during Mhp infection (64). Before and after Mhp infection, the high throughput cDNA microarray assays were used to evaluate the PAMs responses and found some cell behaviors were changed significantly, which could be clustered into inflammatory response, apoptosis, anti-apoptosis, programmed cell death, defense response, signal transduction, and pattern recognition receptors (PRRs) activation (65). PAMs can express Toll-like receptors (TLRs), and the TLR2-TLR6 dimeride can recognize the PAMPs from Mhp, thereby activating the classical Toll/IL-1 receptor signal pathway (66, 67). The up-regulation of TLR2 and TLR4 can also increase the level of IgA in mouse LDC/B cell coculture model (68). Besides, Mhp infection improves the level of proinflammatory cytokines like TNF-α, IL-1β, and IL-6 in PAMs (69, 70), which can be regulated by activator protein-1, nuclear factor κB (NF-κB) and mitogen-activated protein kinase (71, 72).

The regulation of cytokines can also be mediated through other signaling pathways. The cytochrome P450 1A1 (CYP1A1) was down-regulated in lung tissues during Mhp infection; further studies found that the overexpression of CYP1A1 could activate the PPAR-γ signaling pathway and up-regulated the IL-1β, IL-6, IL-8, and TNF-α levels in Mhp-treated PAMs (70). Moreover, sonicated protein fractions from Mhp could stimulate PAMs to produce higher level of IL-1β, IL-6, TNF-α, COX-2, and inducible NO synthase. Besides, differential genes expression analysis indicated that *Mus musculus* chemokine (C-C motif) ligand3 (Ccl3) and *M. musculus* serum amyloid A 3 (Saa3) were associated with the inflammatory responses (69). In general, PAMs are involved in inflammatory responses during Mhp infection with the excessive production of proinflammatory cytokines both *in vitro* (64, 73) and *in vivo* (52, 74, 75).

#### Mhp Can Induce Apoptosis to PAMs by Nitric Oxide and (ROS) Production

NO is a multifunctional biological molecule produced by NO synthase, studies have reported that some *Mycoplasma* species can induce immunosuppressive effect by excess NO production (69, 76, 77). The LAMPs from Mhp can induce a time-dependent apoptosis to PAMs by NO production, oxidative stress response, and caspase-3 activation (36). In addition, research in Mhp-treated murine alveolar macrophage found that the up-regulation of NO level and proinflammatory cytokines were associated with the activation of NF-κB and three MAPK signaling pathways (64). On the other hand, the increased synthesis level of phosphoribosyl pyrophosphate (PRPP), nicotinamide adenine dinucleotide (NAD), and nucleotide in pathogenic strain Mhp 168 can be regarded as an effective protection method for potential ROS damage to Mhp cells (78).

#### PAMs Can Provide Nucleotides by the Digestion of Mhp Nucleases

Mhp cannot synthesize purines or pyrimidines *de novo* while it is able to assimilate exogenous nucleobases and nucleosides and then synthesizes nucleotides by salvage pathways and interconversions (79). Studies had shown that Mhp can digest extracellular DNA to form biofilm in the respiratory tract and abiotic surfaces, which could be inhibited by nuclease treatment (80). Besides, the multifunctional nuclease Mhp597 had been reported (81), and Mhp597 was able to digest extracellular nucleic acid such as induced macrophage extracellular traps (METs) to synthesize nucleotides (80, 82, 83). Another study found Mhp could assimilate nucleotides more effective from METs than free nucleotides in medium, which suggested a close connection between nuclease degradation and nucleotide transportation (84). In general, PAMs can provide nucleotides and protein synthesis materials for Mhp proliferation, and they may serve as a shelter for Mhp to escape immune attacks (85).

### Adaptive Cellular Immune Responses During Mhp Infection

DCs are the most powerful antigen presenting cells that can activate effector CD4^+^ T cells, cross-present antigens to CD8^+^ T cells, and stimulate B cells to produce antibodies (86). DCs are widely distributed among respiratory tracts and have a high density above and below the basement membrane of the tracheal epithelium, which allows DCs to efficiently ingest, process, and present antigens (87). Studies showed that the quantity of SIgA positive cells, T cells, SLA-II-DR^+^CD11b^+^ DCs, and SLA-II-DR^+^SWC3a^+^ DCs decreased obviously during Mhp infection (88). In addition, the level of IL-12 and interferon γ (IFN-γ) was also down-regulated, which indicated that DCs preferred Th2 immune response (89). Another study evaluated the cytokines production of bone marrow–derived DCs (BMDCs) in a coinfection model, and reported the lower IL-12 production of Mhp infection and higher IL-10 level with the coinfection of *M. flocculare* and Mhp (90). Vaccine strain Mhp 168-L treated BMDCs can activate T-cell proliferation effectively, while the up-regulation of IL-10 and down-regulation of IFN-γ may limit its effect (91).

Previous studies evaluated the capability of antibody production by peripheral blood mononuclear cells (PBMCs) and found that older pigs possessed a higher antibody level during Mhp infection (92). Follow-up studies reported Mhp infection could induce apoptosis to PBMCs by ROS and NO production, which were related to the Bax/Bcl-2, p38 MAPK signaling pathway and caspase activation (93). When PBMCs were treated with heat-killed Mhp cells, the proinflammatory cytokines (TNF-α, IL-1β, IL-8, and IL-18) and anti-inflammatory cytokine (IL-10) were up-regulated; at the same time, the level of antigen-specific IFN-γ and IL-10 were gradually decreased during infection (74). These results indicated that Mhp could regulate immune responses by inducing several cytokines secretion and cause immunosuppression with disease development.

### Humoral Immunity for Mhp Infection With SIgA Production and IgG1/IgG2 Bias

The mucosal surface of the respiratory tract is the first defense line to prevent pathogens infection and eliminate microorganisms (bacteria, virus, parasites, and fungi) or foreign bodies by producing active components like SIgA (94, 95). On the other hand, mucosal immunity is an important sign of Mhp infection and could be improved by cathepsin L, which should be considered into the medication development (96).

The deviation of IgG subclass during Mhp infection indicates a Th1/Th2 response bias, in general, the IL-12 is related to the Th1 immune response, the IL-4 and IL-6 are related to the Th2 immune response, while the specific immune type that Mhp induced remains ambiguous (97). Studies found a lower production of IL-12 during Mhp infection, which indicated that the immune system preferred the Th2 immune response (90). Some studies also reported that Mhp could induce a higher level of IgG1 in local and systemic immune responses, which could stimulate the differentiation of naive Th1 cells to Th2 cells (74). On the contrary, some experiments reported the secretion of IgG2 dominated the humoral immune responses, and indicated Mhp could firstly induce the cell-mediated immunity (97–99). In addition, Mhp was thought to exist only extracellularly and adhere to the surface of respiratory tract (60), while recent studies have found that Mhp can also survive inside the epithelial cells (100). Meanwhile, the Th1 immune response can resist the invasion of intracellular pathogens, indicating that Mhp can induce cellular immune response firstly (98).

## New Strategies for Next-Generation Vaccines Development Against Mhp Infection

At present, vaccination (inactivated/attenuated vaccines) is a commonly used and effective method to prevent Mhp infection, while these vaccines provide partial protection and cannot cure MPS or eliminate Mhp from farms or bodies completely. Vaccines preparation need excellent strains with high immunogenicity and adequate supply of bacteria, besides, the product price, immunization efficiency and vaccination method also influence the popularization of such vaccines. In order to improve the immunogenicity and biological characteristics of Mhp strains, the self-replicating plasmids with the origin of replication (*oriC*) sequences from Mhp could be useful genetic engineering vectors for strain improvement, which had expressed EGFP in Mhp cells successfully (85, 101). On the other hand, genome-scale metabolic engineering techniques provided more information for the optimization of fermentation process, studies had reported that the addition of pyruvate could increase Mhp growth rate in special fermentation experiment (102).

The genetically engineered vaccines are still under development, which mainly includes adhesion factor-based single antigen vaccines, multiple antigens combined vaccines, and multiple disease combined vaccines (5). Feasibility phase of vaccine development depends on the effective antigens selection, expression system, and experimental batch proof of concept, which not only wastes time but also extends the research and development cycle (103). On the other hand, genomic variability and Post-translational protein processing will increase the risk of these vaccine development, and may further reduce the general applicability.

To improve the feasibility of novel vaccine development, the computer-aided vaccinology can provide some new strategies and methods. For example, bacterial pan genome analysis tool (BPGA) can evaluate the genomic diversity and classify the core (conserved), accessory (dispensable), and unique (strain-specific) genes of different strains, which can be used for the choice of conserved and dominant immunogens (104). On the other hand, reverse vaccinology can also provide novel framework for the development of multi-epitope vaccines, which had been used to prevent the infection of bacteria (105), virus (106), and parasite (107).

## Discussion

Mhp is an important respiratory pathogen that can cause MPS, and brings enormous economic losses worldwide. MPS can develop into a recessive disease with the immune defense and other treatments; the isolation of Mhp from other tissues has been reported, which indicates that Mhp can turn into an internal organ and exist within its host without causing disease (27, 108–110). Mhp infection decreases host immunity and induces significant apoptosis or dysfunction to immune cells, as well as increasing the secondary viral or bacterial infection risk.

With the development of modern swine production goes to larger-scale and intensive, coinfections of Mhp and other pathogens are becoming common and serious. Porcine circovirus type 2 (PCV2) infection is immunosuppressive by damaging lymphoid tissues; the coinfection of Mhp and PCV2 contributes to a range of polymicrobial disease syndromes (111). What is more, the coinfection of Mhp and reproductive and respiratory syndrome virus (PRRSV) is generally considered to be universal. The quantitative microbial ecology analysis of microbiota was performed in bronchoalveolar lavage fluid (BALF) from PRRSV-infected pigs, and found the dominant bacterial groups were *Haemophilus parasuis* and *Mycoplasma hyorhinis*, which indicated that the pathological importance of *Mycoplasma hyorhinis* had been underestimated (112). Earlier research pointed out that Mhp infection could increase the lung susceptibility to *Pasteurella multocida* (113). Particularly, Mhp plays a leading role during the adhesion of *Pasteurella multocida* type A, which can provide binding sites on the epithelial cell surface (114). In addition, *Actinobacillus pleuropneumoniae* infection can cause Mhp-infected pigs to show more severe respiratory symptoms (115). In general, coinfection of Mhp and other pathogens aggravates the clinical symptoms and increases the mortality. Therefore, the development of appropriate therapeutic plans and multiple pathogens combined vaccines are crucial in the actual disease treatment.

**Figure 3:**
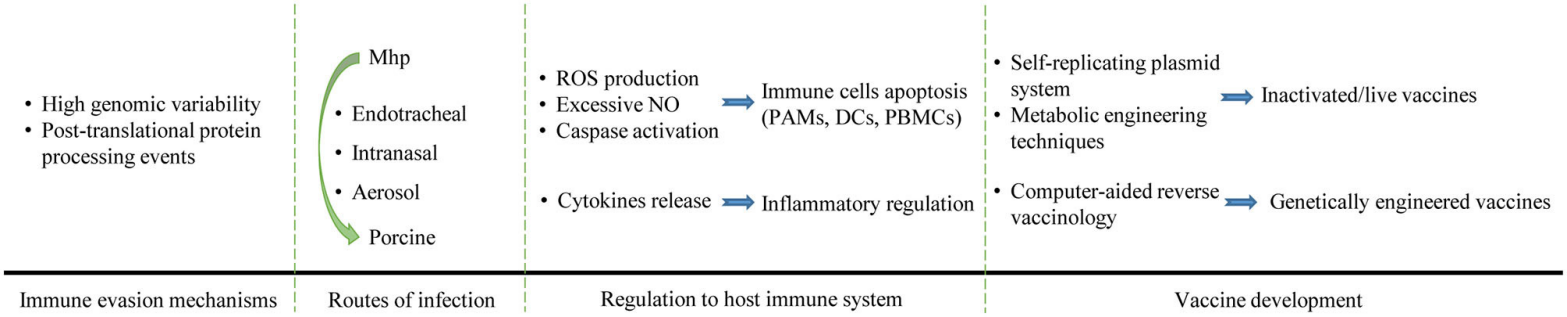
The interaction between Mhp and pig immune system, and new strategies for next-generation vaccine development. Genomic variability and Post-translational protein processing events increase the immune evasion capability of Mhp. Mhp can invade organisms by endotracheal, intranasal, or aerosol. In the interaction between Mhp and the host immune system, Mhp can regulate the inflammatory responses by stimulating the release of cytokines. Mhp can also induce apoptosis to immune cells by ROS production, excessive NO release, and caspase activation. For vaccine development, self-replicating plasmid system and metabolic engineering techniques can be used for strain improvement, and the computer-aided reverse vaccinology is effective to increase the feasibility of novel vaccine development.

The main contents of this article are briefly summarized in Figure 3. Genomic variability and Post-translational protein processing complicate the Mhp cell surface antigens and virulence factors appearance, which enhance the immune evasion of Mhp and make the treatment of MPS more difficult. Protein cleavage can produce more functional motifs and make excess epitopes exposed, which can promote disease development and increase the immune burden. Mhp can invade organisms by endotracheal, intranasal or aerosol. In the interaction between Mhp and the host immune system, Mhp can regulate the inflammatory responses by stimulating the release of cytokines. Mhp can also induce immune cells apoptosis by ROS production, excessive NO release and caspase activation, and increases the risk of coinfection with other pathogens. For vaccine development, the self-replicating plasmid system and metabolic engineering techniques can be used for strain improvement, and the computer-aided reverse vaccinology is effective to increase the feasibility of novel vaccine development.

Mhp is an important pathogen that leads to MPS and causes major economic losses to pig industry, some commercial vaccines are already available, while the limited immune protective effect is unable to eliminate Mhp from farms or bodies. The balance between immune evasion of the pathogen and immune defense of the host determines the disease development, and based on the current research status, we proposed the genomic variability and Post-translational protein processing are important immune evasion mechanisms of Mhp, but it still needs direct concept proof and deeper research on the mechanisms of immune evasion. In order to reduce the adverse effects of immune evasion on vaccine development and increase the general applicability of vaccines, some novel research methods, such as metabolic engineering techniques and computer-aided reverse vaccinology, can improve the feasibility of novel vaccines as well as reducing the research and development cycle. Vaccine development based on reverse vaccinology has been applied to pathogens with immune evasion ability like COVID-19 (116), while the practicality of these technologies still needs further exploration. On the other hand, Mhp infection can impair host immunity by ROS production, excessive NO release, and caspase activation. In order to strengthen immune responses, certain adjuvants can be used as a component in the vaccine formula. What's more, the use of appropriate pharmacotherapy to improve the host immunity, especially mucosal immunity, is another attractive treatment method (96), which can be regarded as an assistant method for vaccine development. Reducing the adverse effects of immune evasion and enhancing host immunity are critical directions for vaccine development and medicine research and unraveling the immune evasion mechanisms and pathogenesis of Mhp will pave the way for the development of new therapeutics for MPS.

## Author Contributions

GL and YH conceived the idea. GL wrote the manuscript. EO drew the Figures 2, 3 as well as the grammar and spell checked the manuscript. JinS collected the data and plotted Figure 1. JianS, JC, YW, and YH provided suggestions for the outline and modified the article. All authors read and approved the final version of the manuscript.

## Conflict of Interest

JS was employed by Zhejiang Hom-Sun Biosciences Co., Ltd. The remaining authors declare that the research was conducted in the absence of any commercial or financial relationships that could be construed as a potential conflict of interest.
